# Using combined ROC curves to improve the diagnostic usefulness of glutaminase, prostaglandins, and 8-isoprostane as biomarkers of autism spectrum disorders;Role in the Glu-GABA-Gln cycle

**DOI:** 10.1186/s12868-026-01020-7

**Published:** 2026-06-24

**Authors:** Afaf El-Ansary, Hanan A. Alfawaz, Manan Alhakbany, Ramesa Shafi Bhat, Geir Bjørklund, Laila Y. Al-Ayadhi

**Affiliations:** 1 Autism Research and Treatment Centre, King Saud University, P.O. Box 2925, Riyadh, 11461 Saudi Arabia; 2 Department of Food Science and Nutrition, College of Food & Agriculture Sciences, King Saud University, P.O. Box 22452, Riyadh, 11495 Saudi Arabia; 3 Department of Physiology, Faculty of Medicine, King Saud University, P.O. Box 2925, Riyadh, 11461 Saudi Arabia; 4 Department of Biochemistry, College of Science, King Saud University, P.O. Box 22452, Riyadh, 11495 Saudi Arabia; 5 Council for Nutritional and Environmental Medicine, Mo i Rana, Norway

**Keywords:** Austism spectrum disorder, Glutaminase, 8-isoprostane, Prostaglandin E2, Receiving operating characteristic

## Abstract

Due to delayed symptoms and dependence of behavioral assessment, early diagnosis of autism spectrum disorder remains challenging. Identification of multivariate biomarker for the etiological mechanisms of ASD may enhance diagnostic accuracy. Multivariable logistic regression combines many predictors into a single risk score (linear predictor), resulting in an optimised ROC curve that enhances diagnostic accuracy over individual markers. The method comprises modelling a binary result, determining the likelihood, and visualising ROC based on the projected probabilities, which often improves individual marker AUCs. In the present study a diagnostic performance for a biomarker panel reflecting glutamatergic dysfunction, oxidative stress, and neuroinflammation was evaluated. Plasma levels of glutaminase, 8-isoprostane, and prostaglandin E₂ (PGE₂) obtained from 44 children with ASD and 40 age-matched controls were evaluated using receiver operating characteristic (ROC) analysis, both individually and in combined ROC models. Glutaminase showed significant negative correlations with both 8-isoprostane and PGE₂, whereas a positive correlation was observed between 8-isoprostane and PGE₂. All the three-biomarker showed good diagnostic performance for ASD on its own with statistically significant (*p* = 0.001) values of AUC of 0.830 for glutaminase, AUC of 0.815 for 8-Isoprostane and AUC of 0.818 for PGE₂. However combined ROC modeling substantially improved diagnostic accuracy by achieving high apparent discriminative performance with AUC value of 0.977 with 92.3% sensitivity and 100.0% specificity. In conclusion, the diagnostic usefulness of independent glutaminase, 8-isoprostane, and prostaglandin E₂ (PGE₂) biomarkers may be enhanced by combining ROC. Combined markers show strong apparent discriminating power in a case-control method, but estimates are biassed towards optimism and are not diagnostic. Comprehensive assay validation, calibration, and clinically representative cohorts (including females and relevant differentials) are required for replication.

## Introduction

Autism spectrum disorder (ASD) is a developmental disability characterized by difficulties with social interaction and communication, as well as limited interests or repetitive behaviors. ASD is characterized as a diverse condition, with significant variation in the type and severity of causes, symptoms, and levels of care required across ASD patients (1). In addition to developmental surveillance, the American Academy of Pediatrics recommends that all children between the ages of 18 and 24 months be tested for ASD (Hyman et al., 2020, [23]). Additional screening may be required if a child is at high risk for ASD or has signs and symptoms.

Although therapeutic interventions work best when initiated early in infancy, the diagnosis of ASD is frequently postponed, partly due to the diagnostic’s reliance on identifying abnormal behaviors that might not manifest until the condition has advanced considerably.

Biomarkers that might identify children at risk before symptoms manifest, enable early diagnosis, validate behavioural observations, classify patients into subgroups, and predict treatment response would be of great help [12].

Because the total volume of Cerebrospinal fluid (CSF) averages between 150 and 270 mL, it is replenished around four times per day [30, 15]. Plasma as a complex body fluid containing proteins, peptides, lipids and metabolites that reflect physiological activity and pathology in various body organs, including the CNS. In humans about 500 ml of CSF is absorbed into blood daily, making blood a suitable source of brain disease biomarkers [16, 18, 22].

Glutaminase transforms glutamine into glutamate, the major excitatory neurotransmitter that regulates synaptic glutamate availability and controls the astrocytic-neuronal glutamate-glutamine cycle. Hamed et al. [14] studied glutaminase, glutamine synthetase, and glutamate decarboxylase autoantibodies in a subset of autistic people, indicating a direct immune-mediated disruption of excitatory-inhibitory neurotransmission and supporting the idea that enzymatic imbalance contributes to ASD neurobiology. Glutamine deprivation or glutaminase inhibition also stimulated the production of reactive oxygen species [27].

Prostaglandin E2 (PGE2), as a product of cyclooxygenase 1 &2, has been shown to modulate neuronal activity and synaptic function. It exerts its effects through four distinct G-protein-coupled receptors (EP1-EP4), which are differentially expressed in various brain regions and are involved in regulating processes such as neurotransmitter release, synaptic plasticity, and neuroinflammation [21, 2]. In the context of neurodevelopmental disorders like ASD, aberrant prostaglandin signalling has drawn increased interest due to its role in brain development and function [11].

Research demonstrates that combining biomarkers associated with lipid metabolism and neuroinflammation results in a greater Area Under the Curve 8-isoprostane, which is the most sensitive indicator of oxidative stress and a widely used marker, has been discovered to play an important role in some neurological disorders among which is ASD [24, 28, 9].

Numerous studies have demonstrated the importance of the relationship between neuroinflammation and oxidative stress. It has been demonstrated that prevalent oxidative stress promotes chronic inflammation [13, 17]. High ROS concentrations can activate signaling pathways and cause excessive release of pro-inflammatory cytokines and chemokines, promoting more ROS formation in a vicious cycle [13].

According to research, combining biomarkers associated with lipid metabolism and neuroinflammation results in a higher Area Under the Curve (AUC) in ROC analysis, indicating improved sensitivity and specificity for autism detection [1]. A panel of glutaminase, 8-isoprostane, and prostaglandin E₂ (PGE₂) biomarkers may provide a comprehensive approach to early diagnosis of ASD by focusing on three key pathogenic mechanisms: oxidative stress, chronic inflammation, and excitatory/inhibitory imbalance.

While prostaglandins E2 can affect inflammation and cell survival in addition to other mediators, glutamine, a glutaminase substrate, functions as a primary antioxidant precursor (via glutathione) to counteract the oxidative damage induced by isoprostanes [36, 37].

This study examines how glutaminase, 8-isoprostane, and prostaglandin E₂ (PGE₂) work together to boost discriminative ability in people with ASD compared to controls. The diagnostic utility is not claimed.

## Materials and methods

### Participants

The study included 44 children diagnosed with autism spectrum disorder (ASD) and 40 age-matched control children. Children with ASD were enrolled from the Autism Research and Treatment Center and Well baby Clinic at King Khalid University Hospital, while control participants were recruited from the Pediatric Laboratory Center of King Saud Medical City, Riyadh, Saudi Arabia. This work was approved by the King Saud University King Khalid Hospital Ethics Committee. In compliance with the guidelines set forth by the King Saud University King Khalid Hospital Ethics Committee, each subject’s parents gave written consent.

ASD diagnoses were established using internationally recognized diagnostic tools, including the Autism Diagnostic Interview–Revised (ADI-R), Autism Diagnostic Observation Schedule (ADOS), and the Developmental, Dimensional and Diagnostic Interview (3DI). The mean age of children in the ASD group was 7 ± 4 years and the control group had a mean age of 7 ± 3 years.

All Participants (Autism and Control) were excluded if they had a history of neurological disorders (such as seizures), psychiatric illnesses (including bipolar disorder), Autoimmune, inflammatory or chronic medical conditions. Children with diagnosed endocrine, cardiovascular, respiratory, hepatic, renal, or other systemic diseases were also excluded. Participants undergoing therapies such as gluten-free/casein-free (GFCF) diets, ketogenic diets (KD), and supplements were also excluded.

The study protocol was approved by the Institutional Review Board of the College of Medicine, King Saud University (No. 22/0122/IRB) and adhered to the Guidelines for Research on Human Subjects of the Health Sciences Colleges. Each participant’s parents gave written consent to participate in line with the guidelines set forth by the King Saud University King Khalid Hospital’s Ethics Committee.

### Blood sample collection

Blood samples were obtained after an overnight fast from children with ASD and controls by a trained laboratory professional. Blood was collected into 3-mL tubes containing ethylenediaminetetraacetic acid (EDTA). Samples were immediately centrifuged at 3000 × g for 20 min at 4 °C. Plasma was separated, aliquoted to prevent repeated freeze–thaw cycles, and stored at − 80 °C until analysis.

### Biochemical analyses

#### Determination of glutaminase

Plasma glutaminase levels were measured using an enzyme-linked immunosorbent assay (ELISA) kit obtained from MyBioSource (San Diego, CA, USA). The assay employs a quantitative sandwich enzyme immunoassay technique and was performed according to the manufacturer’s instructions. Microtiter plates were pre-coated with an antibody specific for human GLS. The enzyme–substrate reaction was terminated by the addition of sulfuric acid solution, and absorbance was measured spectrophotometrically at 450 ± 2 nm. The detection range of the assay was 0.312–20 ng/mL.

#### Determination of 8-isoprostane

Plasma levels of 8-isoprostane were determined using a non-radioactive ELISA kit obtained from MyBioSource (San Diego, CA, USA). This assay utilizes a quantitative sandwich enzyme immunoassay technique and was performed in accordance with the manufacturer’s protocol. The minimum detectable concentration of human 8-isoprostane was typically less than 19.5 pg/mL.

#### Determination of prostaglandin E₂

Plasma PGE₂ concentrations were measured using a commercially available ELISA kit obtained from USCN Life Science (Wuhan, China). This assay uses the competitive inhibition enzyme immunoassay method, in which a monoclonal antibody specific for human PGE2 was pre-coated on a microplate. The minimum detectable concentration was typically less than 1.78 pg/mL.

### Statistical analysis

Statistical analyses were carried out using IBM SPSS Statistics software (version 22.0; IBM Corp., Armonk, NY, USA), GraphPad Prism program version 9.5 and MedCalc Version 22.019. Differences between two independent non-parametric groups were evaluated using the Mann–Whitney U test. A p-value ≤ 0.05 was considered statistically significant. Relationships between variables were assessed using Spearman’s rank correlation coefficient (R) with graphic representations using Correlation matrix (Heat map), with values ranging from − 1 to + 1, indicating inverse and direct associations, respectively.

In order to predict the likelihood of having an ASD diagnosis, the possible biomarkers are included into a single regression equation to create the combined ROC model using logistic regression. The area under the Receiver operating characteristic ROC curve (ROC-AUC) was calculated using a non-parametric approach. An AUC value close to 1.0 suggests high discriminative capacity, whereas an AUC of 0.5 implies no diagnostic importance. Sensitivity and specificity were calculated to establish biomarker accuracy in differentiating autistic patients from controls. The higher specificity found with combination biomarkers shows that a multi-marker panel may provide better diagnostic performance than single biomarkers.

## Results

**Fig. 1:**
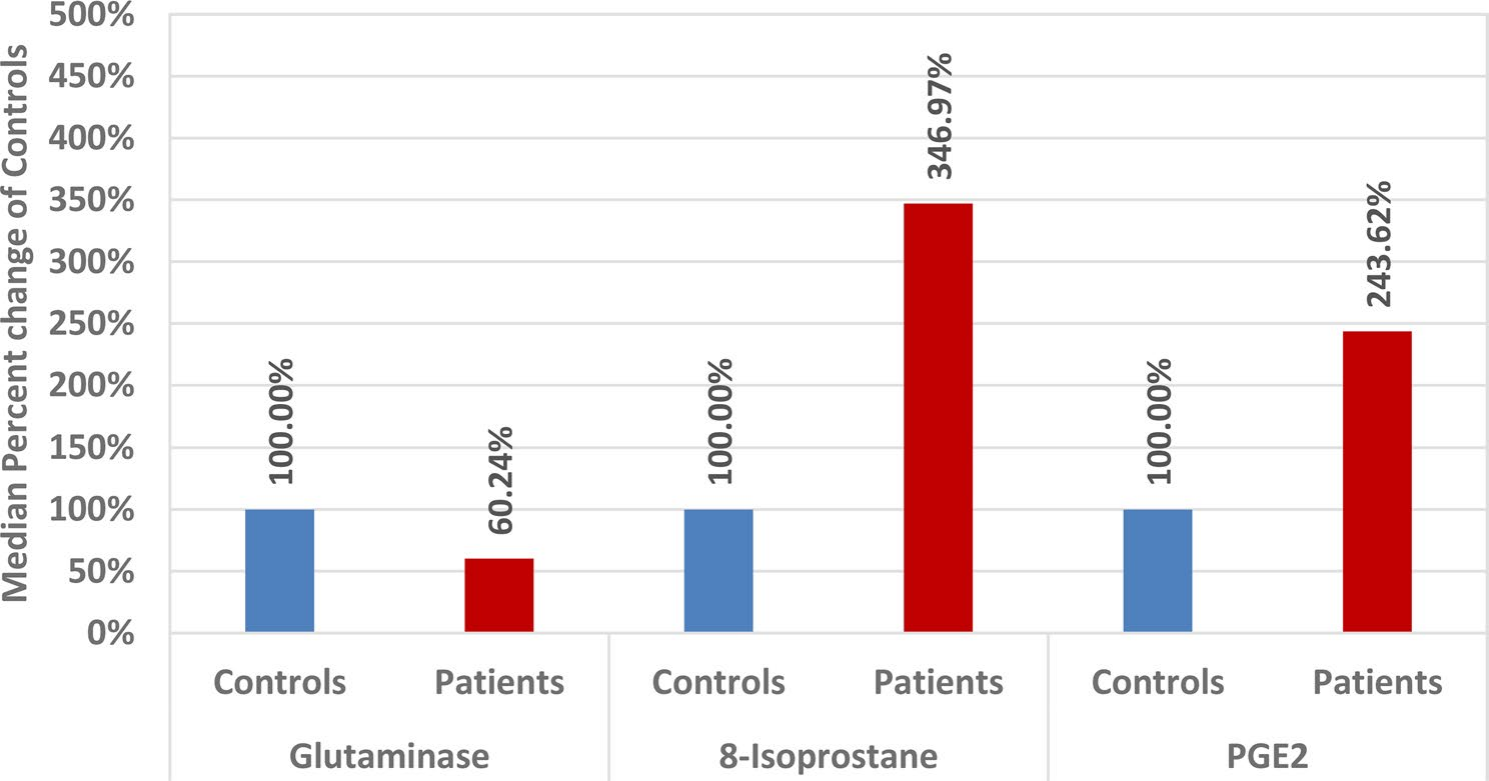
Percent change of Median for (Patients) according to (Controls) group in the following parameters

Levels of all measured biomarkers were compared between ASD patients and control groups and are presented in Table 1. Significant differences were observed between ASD patients and control participants for all three measured biomarkers (Table 1). Glutaminase levels were significantly reduced in the ASD group compared with controls (*p* = 0.001), representing a decrease to approximately 60% of control values. In contrast 8-isoprostane concentrations were significantly higher in patients compared with controls (*p* = 0.001) corresponding to a 3.5-fold increase. Similarly, PGE₂ levels were significantly increased in the ASD group (*p* = 0.001), reflecting a 2.4-fold elevation. Percent changes relative to control medians are illustrated in Fig. 1.

**Table 1:** Comparison between (Patients and Controls) groups for the following parameters

Parameters	Groups	Min.	Max.	Mean ± S.D.	Percent change	*P* value^#^
Glutaminase	Controls	0.09	0.40	0.18 ± 0.08	100.00	0.001
	Patients	0.06	0.22	0.11 ± 0.04	60.24	
8-Isoprostane	Controls	19.02	29.16	24.25 ± 2.58	100.00	0.001
	Patients	0.00	134.52	84.15 ± 42.04	346.97	
PGE2	Controls	1.46	9.35	4.42 ± 1.70	100.00	0.001
	Patients	0.00	20.25	10.76 ± 5.71	243.62	

^#^ *Calculated by Mann-Whitney Test (Non-Parametric data)*

**Fig. 2:**
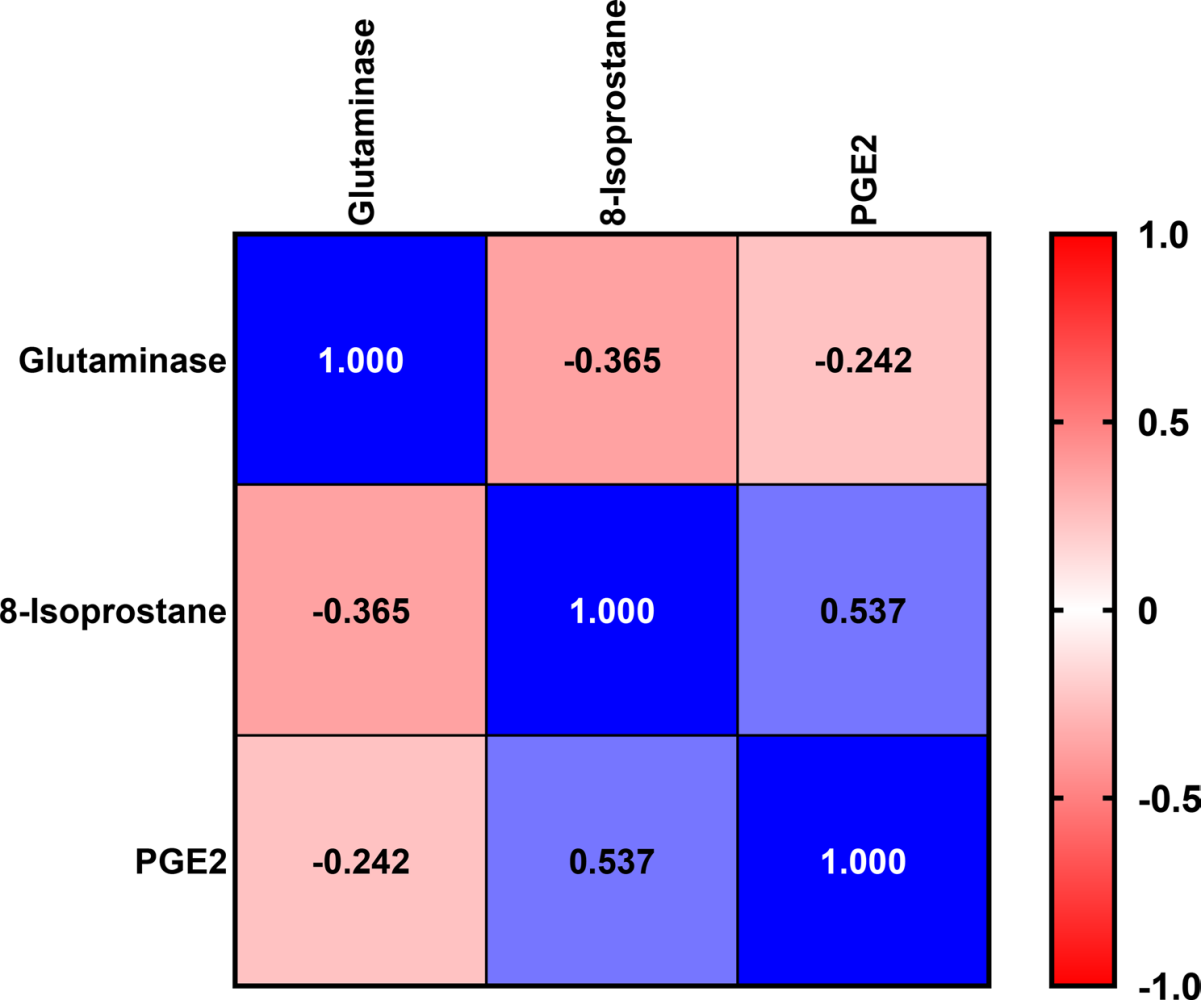
Heat map for the following parameters using non-parametric Spearman correlation for (All groups)

Spearman correlation analysis of the measured biomarkers in Table 2 revealed significant associations among the three biomarkers. Glutaminase levels showed a significant negative correlation with both 8-isoprostane (*p* < 0.01) and PGE₂ (*p* < 0.05). In contrast, a significant positive correlation was observed between 8-isoprostane and PGE₂ levels (*p* < 0.01). A heat map of the three parameters using Nonparametric Spearman’s correlation across all groups is shown in Fig. 2.

**Table 2:** Correlations between the three measured variables using Spearman Correlation

All Groups	Glutaminase	8-Isoprostane	PGE2
Glutaminase	1.000	-0.365**	-0.242*
8-Isoprostane	-0.365**	1.000	0.537**
PGE2	-0.242*	0.537**	1.000

** Correlation is significant at the 0.01 level

*Correlation is significant at the 0.05 level

**Fig. 3:**
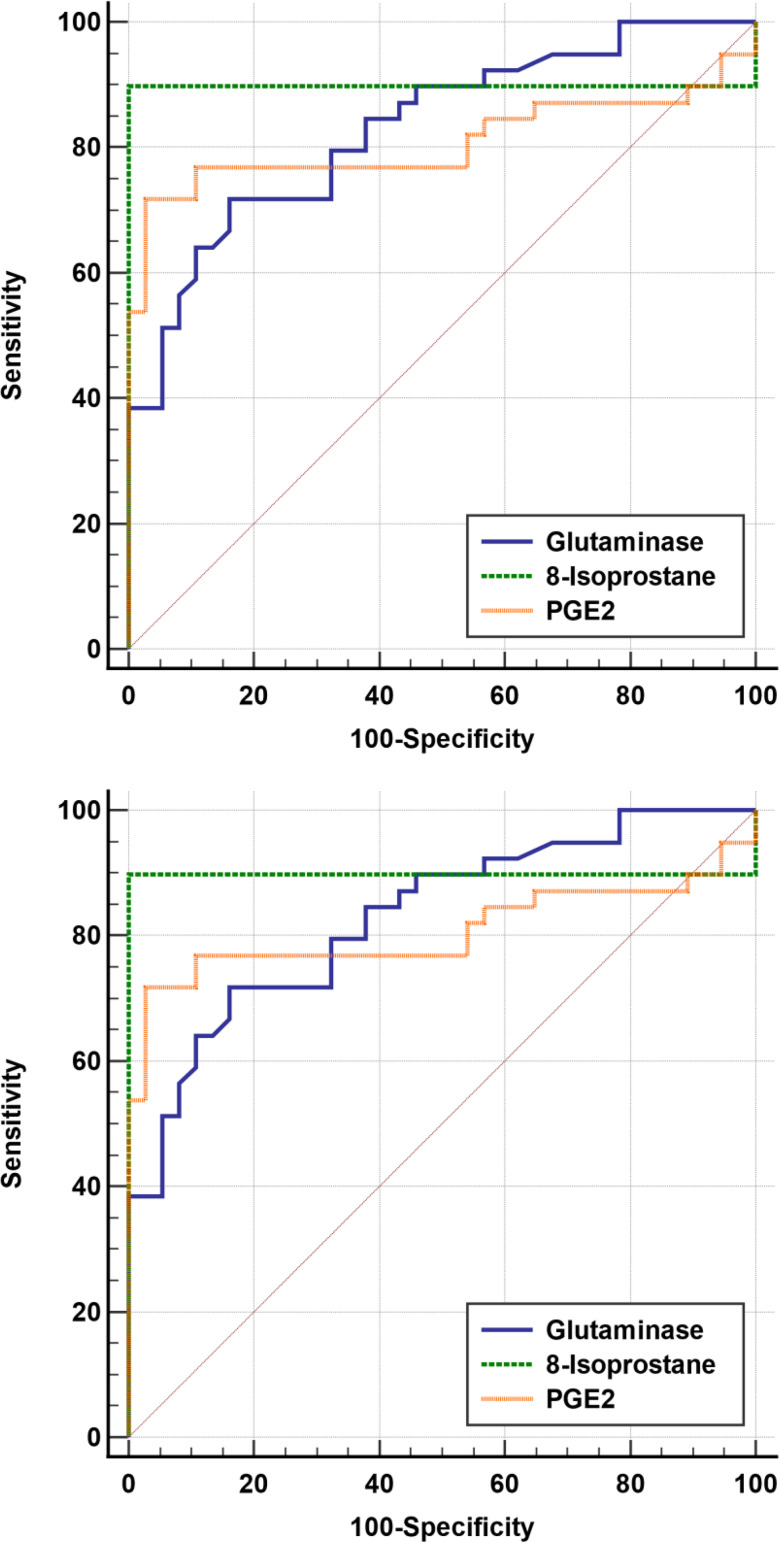
ROC Curve of the following parameters for (Patients) according to (Controls) group as a reference group

Receiver Operating Characteristic (ROC) curve analysis presented in Table 3; Fig. 3 demonstrated that each biomarker individually exhibited acceptable discriminatory ability for differentiating ASD patients from controls. Glutaminase yielded an AUC of 0.830 with a sensitivity of 71.8% and specificity of 83.8% at the optimal cut-off value. 8-Isoprostane demonstrated an AUC of 0.815, achieving a sensitivity of 81.5% and a specificity of 100.0%. PGE₂ also showed good diagnostic performance, with an AUC of 0.818, sensitivity of 72.3%, and specificity of 97.3%. All AUC values were statistically significant (*p* = 0.001).

**Table 3:** ROC Results for (Patients) according to (Controls) group as a reference group

Parameters	AUC	95% CI	Cut-off value	Sensitivity %	95% CI	Specificity %	95% CI	*P* value
Glutaminase	0.830	0.740–0.920	0.116	71.8%	55.1–85.0	83.8%	68.0–93.8	0.001
8-Isoprostane	0.815	0.711–0.918	55.049	81.5%	68.6–90.7	100.0%	90.5–100.0	0.001
PGE2	0.818	0.719–0.918	7.302	72.3%	57.4–84.4	97.3%	85.8–99.9	0.001

**Fig. 4:**
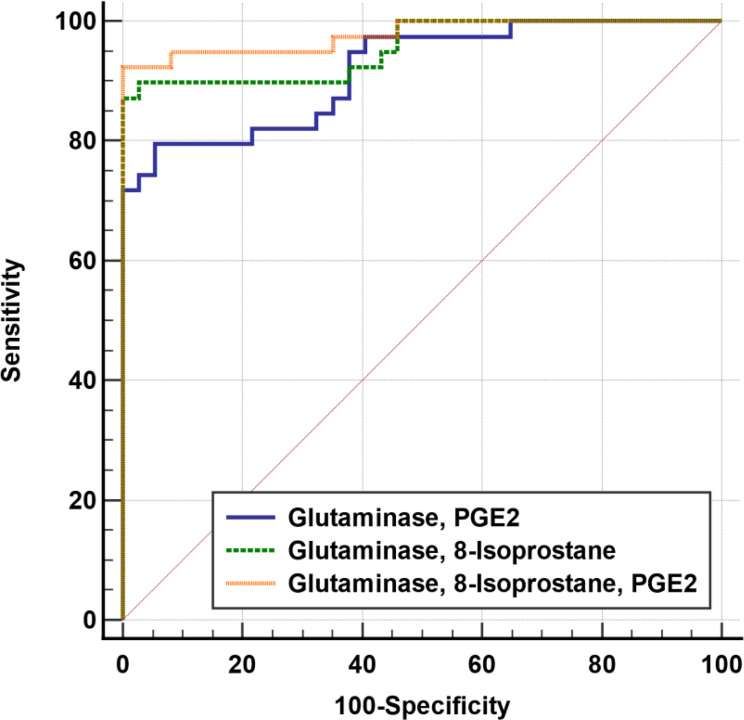
Combined ROC Curve of the following parameters for (Patients) according to (Controls) group as a reference group

To assess whether combining biomarkers improved discriminatory performance, combined ROC analyses were performed as presented in Table 4; Fig. 4. The combination of glutaminase and PGE₂ resulted in an AUC of 0.918 with a sensitivity of 79.5% and specificity of 94.6%. Combining glutaminase with 8-isoprostane further increased the AUC to 0.955 achieving a sensitivity of 87.2% and specificity of 100.0%. The highest discriminatory performance was observed when all three biomarkers were combined, yielding an AUC of 0.977 with a sensitivity of 92.3% and specificity of 100.0%.

**Table 4:** Combined ROC Results of the following parameters for (Patients) group according to (Controls) group as a reference group

Parameters	AUC	95% CI	Sensitivity %	95% CI	Specificity %	95% CI	*P* value
Glutaminase, PGE2	0.918	0.857–0.978	79.5%	63.5–90.7	94.6%	81.8–99.3	0.001
Glutaminase, 8-Isoprostane	0.955	0.910-1.000	87.2%	72.6–95.7	100.0%	90.5–100.0	0.001
Glutaminase, 8-Isoprostane, PGE2	0.977	0.947-1.000	92.3%	79.1–98.4	100.0%	90.5–100.0	0.001

## Discussion

The present study demonstrates a significant alteration in glutamatergic metabolism and redox– inflammatory tone in patients compared with controls, with reduced plasma glutaminase and marked elevations in 8-isoprostane and PGE₂ (Table 1; Fig. 1).

These findings emphasise the importance of abnormal glutamate-GABA-glutamine cycles, glutamate-mediated excitotoxicity, and downstream oxidative/inflammatory cascades in detecting individuals with ASD. While all biomarkers had acceptable AUC values, combining the three variables considerably enhanced ASD individuals’ discrimination from control.

The significant decrease in glutaminase (Table 1; Fig. 1) could find support in the work of Chenhui et al. (2023) which examined a previously published transcriptome dataset and found reduced glutaminase transcript levels in the peripheral blood or postmortem brain of various cohorts of ASD patients relative to matched controls. Furthermore, Gls1CamKIIa-Cre mice, a conditional transgenic mouse with glutaminase deficiency in forebrain glutamatergic neurons at the postnatal stage, showed ASD behavioral abnormalities such as reduced social interaction and repetitive behaviors, as well as impaired spatial learning and memory.

In ASD, glia-mediated inflammation can be upregulated by oxidative stress, creating a vicious cycle. Unnisa et al. [34] found that when there is inflammation, reactive glial cells cause autism and synaptic dysfunction. The development of autism, may be influenced by dysfunction or changes in microglia’s ability to carry out physiological and defensive tasks (such as synaptic clearance failure or aberrant microglial activation).

Table 2; Fig. 2 show a significant negative correlation (*P* < 0.01) between 8-isoprostane, a marker of oxidative stress, and PGE2, a marker of inflammation (*P* < 0.05) in one hand and glutaminase in the other hand, indicating that lower glutaminase activity correlates with increased oxidative stress and neuroinflammation.

Several investigations have found higher isoprostane levels in blood samples from children with ASD [5, 6, 33]. The kinds of biomarkers and application techniques that can more accurately determine the degree and the role of lipid peroxidation in ASD individuals now require more research.

Abnormalities in the PGE2 signaling pathway have been linked to neurodevelopmental diseases like ASD by increasing the amplitude of calcium variation in neuronal growth cones, which affects neurite extension length. As a result, PGE2 may influence intracellular calcium dynamics in differentiated neuronal cells, thereby affecting nervous system development in its early stages [19]. PGE2 signaling has been suggested as an essential regulating factor between oxidative stress and ASD.

Although glutaminase expression was found to be reduced in the postmortem anterior cingulate cortex of patients with ASD, it is unclear how glutaminase loss generates synaptic impairments and ASD-like behavioral phenotypes [32].

The three variables can be readily linked and connected to synaptic impairment and glutamate excitotoxicity as a signaling pathway related to glutaminase deficiency, elevation of isoprostane as a marker of oxidative stress, and PGE2 as a marker of glial cell activation and neuroinflammation. The recorded negative correlation between glutaminase and 8-isoprostane and PGE2 along with a noticeably increased AUC of combined ROC could help to suggest that performance is relatively better by combining several markers linked to particular signalling pathways (Tables 2, 3 and 4; Figs. 2, 3 and 4).

In order to interpret the observed increase in ROC-AUCs for the three combined variables, it is worth noting that cerebral oxidative glucose metabolism is strongly correlated with the activity of the glutamate/GABA-glutamine cycle, a significant metabolic flux in the brain [25, 26]. It is well known that under normal physiological conditions, glutathione (GSH), a major antioxidant, connects to this cycle and improves its function in preventing excitotoxicity and maintaining brain health by using glutamate as a building block and influencing neuronal energy/oxidative balance, especially during stress or injury [29]. However, significantly lower GSH levels in individuals with ASD could play a role in the impairment of the cycle and the reported lower level of glutaminase enzyme [8, 20].

It is interesting to report that prostaglandin E2 (PGE2) can modulate the glutamate-glutamine cycle by influencing glutamate transport and release within the central nervous system (CNS), particularly involving astrocytes. During inflammation, TNFα stimulates glutamate release through prostaglandin E2 in astrocytes, leading to impaired glutamate transport [4]. Cheung et al. [7] suggested that glutamate concentration may be key in determining the pathways of cell death, with higher concentrations preferentially triggering necrosis and lower concentrations leading to apoptosis. Even a transient excess of glutamate can trigger a number of events that eventually cause death.

Impaired GABAergic transmission could be a major contributor to the E/I ratio imbalance that leads to the development of ASD [10]. PGE2 inhibits GABA synapses’ release onto Parvocellular Neurosecretory Cells (PNCs) by activating presynaptic EP3 receptors, which may explain the disinhibition of HPA axis activity during inflammation. Yui et al. [37].

Because astrocytes are critical for removing glutamate from the extracellular space, promoting brain homeostasis, and preventing excitotoxicity, the elevated 8-isoprostane levels reported in the current study can be linked to an impaired glutamate-GABA-Glutamine cycle via the induction of dysfunctional astrocytes [3, 31, 35].

## Conclusion

This study employs ROC curve analysis to determine the predictive value of biomarkers linked with lipid metabolism and the glutamate-GABA-glutamine cycle in children with ASD. It describes how glutaminase influences oxidative stress and neuroinflammation, as well as its negative connection with lipid-related biomarkers 8-isoprostane and PGE2.

In this dataset, marker combinations produce higher apparent discrimination; nevertheless, estimates are optimism-biased by design and should not be used to assess diagnostic performance. Although peripheral data do not permit inference of causality or CNS origin, the associations are consistent with the involvement of astrocyte-neuron signalling. The validation of the assay will determine its eventual clinical utility.

### Limitations

The current study had certain drawbacks. The study cohort had a small sample size due to difficulties in collecting blood samples from children. Another limitation is that the study was only conducted at the Autism Research and Treatment Centre (ARTC), King Saud University, which may limit its application to all children with ASD. Validation in multi-centre studies is critical. While the age distributions were identical by design, there were variations in clinical characteristics such as familial autoimmunity and allergies. At the time of sample, inadequate information was provided about medications, recent illnesses, antibiotic use, diet, sleep, and gastrointestinal symptoms. Each of these factors can have an impact on circulating proteins and small molecules, perhaps contributing to differences across groups. The lack of female participants in this study may restrict the generalizability of the results, as sex is a significant biological determinant in ASD biomarker research. Due to the limited sample size (44 ASD and 40 controls), ROC modelling and multiple comparisons were exploratory. One significant methodological constraint is the lack of internal validation techniques like split-sample validation or cross-validation.

Therefore, these findings should be viewed as hypothesis-generating and necessitate both longitudinal, adequately powered replication with mechanistic readouts and validation.

## Data Availability

The datasets used and/or analysed during the current study available from the corresponding author on reasonable request.
